# Second-site suppressors of HIV-1 capsid mutations: restoration of intracellular activities without correction of intrinsic capsid stability defects

**DOI:** 10.1186/1742-4690-9-30

**Published:** 2012-04-19

**Authors:** Ruifeng Yang, Jiong Shi, In-Ja L Byeon, Jinwoo Ahn, Jonathan H Sheehan, Jens Meiler, Angela M Gronenborn, Christopher Aiken

**Affiliations:** 1 Department of Pathology, Microbiology and Immunology, Vanderbilt University School of Medicine, Nashville, TN, USA; 2 Department of Structural Biology, University of Pittsburgh Medical School, Pittsburgh, PA, USA; 3 Center for Structural Biology, Vanderbilt University, Nashville, TN, USA; 4 Department of Pathology, Microbiology and Immunology, Vanderbilt University School of Medicine, A-5301 Medical Center North, Nashville, TN 37232-2363, USA

**Keywords:** HIV-1, Capsid, Uncoating, Suppressor mutations, Inhibitor

## Abstract

**Background:**

Disassembly of the viral capsid following penetration into the cytoplasm, or uncoating, is a poorly understood stage of retrovirus infection. Based on previous studies of HIV-1 CA mutants exhibiting altered capsid stability, we concluded that formation of a capsid of optimal intrinsic stability is crucial for HIV-1 infection.

**Results:**

To further examine the connection between HIV-1 capsid stability and infectivity, we isolated second-site suppressors of HIV-1 mutants exhibiting unstable (P38A) or hyperstable (E45A) capsids. We identified the respective suppressor mutations, T216I and R132T, which restored virus replication in a human T cell line and markedly enhanced the fitness of the original mutants as revealed in single-cycle infection assays. Analysis of the corresponding purified N-terminal domain CA proteins by NMR spectroscopy demonstrated that the E45A and R132T mutations induced structural changes that are localized to the regions of the mutations, while the P38A mutation resulted in changes extending to neighboring regions in space. Unexpectedly, neither suppressor mutation corrected the intrinsic viral capsid stability defect associated with the respective original mutation. Nonetheless, the R132T mutation rescued the selective infectivity impairment exhibited by the E45A mutant in aphidicolin-arrested cells, and the double mutant regained sensitivity to the small molecule inhibitor PF74. The T216I mutation rescued the impaired ability of the P38A mutant virus to abrogate restriction by TRIMCyp and TRIM5α.

**Conclusions:**

The second-site suppressor mutations in CA that we have identified rescue virus infection without correcting the intrinsic capsid stability defects associated with the P38A and E45A mutations. The suppressors also restored wild type virus function in several cell-based assays. We propose that while proper HIV-1 uncoating in target cells is dependent on the intrinsic stability of the viral capsid, the effects of stability-altering mutations can be mitigated by additional mutations that affect interactions with host factors in target cells or the consequences of these interactions. The ability of mutations at other CA surfaces to compensate for effects at the NTD-NTD interface further indicates that uncoating in target cells is controlled by multiple intersubunit interfaces in the viral capsid.

## Background

During retrovirus maturation, the viral capsid protein (CA) assembles into a shell, referred to as the capsid, surrounding the viral genomic ribonucleoprotein (RNP) complex. HIV-1 CA consists of 231 residues that fold into two distinct domains connected by a flexible linker. Various surfaces of CA are involved in HIV-1 capsid formation. Interactions between the N-terminal domains (NTDs) form hexamers through an intersubunit NTD-NTD interface, while the C-terminal domains (CTDs) form dimers that connect adjacent hexamers through a CTD-CTD interface [1-5]. The existence of an NTD-CTD interface in the retroviral capsid was originally inferred from studies of the Rous sarcoma virus (RSV), in which two lethal MHR mutations in the CTD were rescued by compensatory mutations in the NTD [6]. In HIV-1, NTD-CTD contacts were detected by hydrogen-deuterium exchange [7] and chemical cross-linking [8], and in structures of CA hexamers and pentamers [9,10].

Following cell entry, the HIV-1 particle releases its core into the host cytoplasm. Subsequently, the core undergoes an uncoating process, which we define as disassembly or dissociation of the viral capsid from the internal RNP complex [11]. The details of HIV-1 uncoating, including the timing, location, and mechanism, are poorly understood. HIV-1 preintegration complexes (PICs) isolated from the cytoplasm several hours after virus entry contain only low levels of CA protein, suggesting that viral uncoating occurs in the cytoplasm prior to nuclear entry [12,13]. Reverse transcription complexes have been isolated at earlier time points, with some of these complexes retaining low levels of CA [14]. Recent studies employing imaging and pharmacologic approaches have suggested that uncoating takes place within a few hours following cell entry, and may be linked to reverse transcription [15,16].

In a previous study, we observed that mutations in CA that result in altering the intrinsic stability of the HIV-1 capsid are associated with impaired infectivity [17]. These capsid stability mutants were competent for viral particle assembly and release and exhibited normal core morphology by electron microscopy, but most exhibited defects in reverse transcription in target cells. One of the mutants, Q63A/Q67A, resulting in unstable capsids *in vitro*, was competent for reverse transcription but impaired for nuclear import [17,18]. Interestingly, PICs recovered from this mutant contained elevated levels of CA and were impaired for integration *in vitro*. Collectively, these studies suggest that uncoating occurs progressively in the cytoplasm and is required for productive reverse transcription, nuclear import, and integration.

Viral determinants besides CA may also be involved in HIV-1 uncoating. A triple-stranded viral DNA structure created during reverse transcription was implicated in uncoating at the nuclear pore. This so-called "DNA flap" structure was shown to be required for nuclear import, but not reverse transcription [19]. Mutations in the HIV-1 matrix protein (MA) can also result in early postentry defects, suggesting that MA also regulates uncoating [20-22].

HIV-1 uncoating may also be modulated by host cell factors. Cellular proteins that may affect uncoating include cyclophilin A and restrictive alleles of TRIM5α [23,24]. However, whether these proteins play a direct role in uncoating remains unknown, owing to a lack of sensitive and validated cell-based assays to study HIV-1 uncoating in target cells and the difficulty in distinguishing functional from nonfunctional particles [25]. A recent study also reported that lysates from activated CD4^+ ^lymphocytes, but not quiescent CD4^+ ^lymphocytes, induce disassembly of isolated HIV-1 cores *in vitro*, suggesting that HIV-1 uncoating may differ in activated vs. resting T cells [26].

Amino acids known to affect HIV-1 capsid stability are located on both domains of the CA protein in varying locations, suggesting that multiple surfaces of the CA protein are involved in stabilizing intersubunit contacts. In the present study, we identified second-site substitutions in CA that restore the ability of HIV-1 mutants containing unstable (P38A) or hyperstable (E45A) capsids to replicate in T cells. Surprisingly, the suppressor mutations rescue HIV-1 infectivity without correcting the intrinsic capsid stability defects associated with the original mutations.

## Results

### Second-site suppressor mutations restore replication to the P38A and E45A mutants

**Figure 1:**
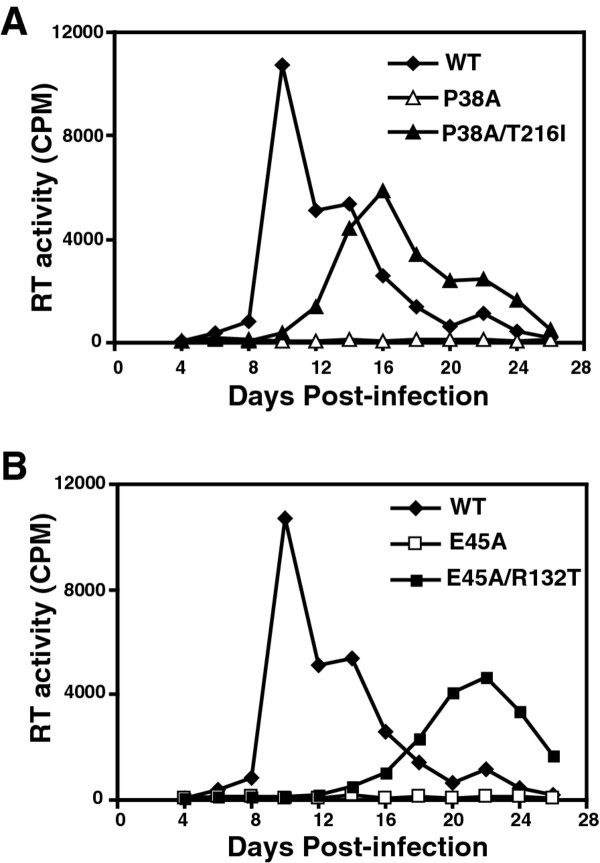
**Rescue of P38A (A) and E45A (B) mutants by second-site mutations T216I and R132T, respectively**. Replication of mutant and wild-type viruses was analyzed in CEM cells. Samples were collected on the days indicated and analyzed for reverse transcriptase (RT) activity. Shown are the average values obtained from duplicate parallel cultures.

To probe the role of domain interactions in maintaining optimal HIV-1 capsid stability, second-site suppressor mutations were isolated by serial passage of P38A and E45A mutant viruses in human CEM T cells. Virus supernatants were harvested immediately following the peak of growth of the mutant virions and were inoculated into fresh cells and the cultures were maintained until the reemergence of HIV-1 in the cultures. To determine whether phenotypic reversion was associated with acquisition of compensatory HIV-1 mutations, DNA was purified from infected CEM cells harvested near the peak of growth. Sequence analysis of PCR products from the P38A culture revealed the presence of a mutation at codon 216 resulting in a substitution of Thr to Ile (T216I) in addition to the original P38A mutation. Sequence analysis of PCR products from the E45A culture revealed the presence of a mutation at codon 132 resulting in a substitution of Arg to Thr (R132T) in addition to the original E45A mutation. No other changes were observed in the PCR-amplified viral sequences. To determine whether these second-site substitutions were responsible for the accelerated growth kinetics of the adapted viruses, the mutations were transferred into the original P38A and E45A mutant viral clones. A replication assay was performed with the resulting double mutant viruses in CEM cells. In contrast to the original P38A and E45A mutants, which failed to replicate in CEM cells within 26 days, the P38A/T216I and E45A/R132T double mutants both replicated, though each was delayed relative to the wild type (Figure 1A, B). These results demonstrate that the respective second-site mutations, T216I and R132T, partially restore the ability of the corresponding P38A and E45A mutant viruses to replicate in CEM cells.

### Second-site suppressor mutations enhance the infectivity of the P38A and E45A mutants in a single-cycle assay

**Figure 2:**
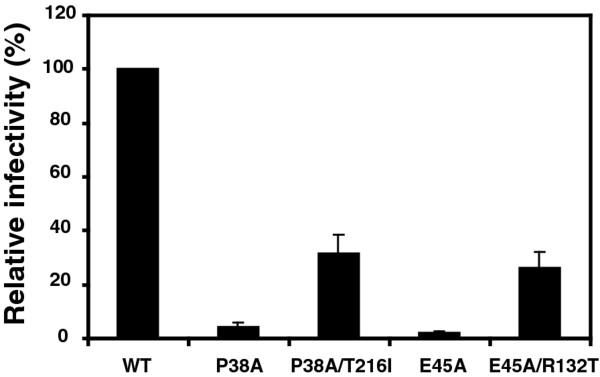
**Rescue of P38A and E45A infectivity defects mutations by second-site mutations T216I and R132T**. Single-cycle infectivity was assayed in HeLa-P4 target cells. Infectivity was determined as the number of infected cells per ng of p24 in the inoculum, and values are expressed as percentage of wild-type HIV-1 infectivity. Results shown are the mean values of three independent experiments, with error bars representing one standard deviation.

Our group previously showed that the P38A and E45A mutants are impaired at an early post-entry stage of infection [17]. Therefore, we sought to determine whether the second-site mutations rescue the P38A and E45A mutant viruses in a single-round reporter assay. This assay, which requires expression of Tat from an integrated provirus, is a useful tool to identify defects in the early stages of infection. Quantitation of virus infectivity using the HeLa-P4 cell line (HeLa-CD4/LTR-*lacZ*) revealed that the T216I and R132T second-site mutations markedly enhanced the infectivity of P38A and E45A, respectively (Figure 2). We also constructed and determined the infectivity of the single mutants T216I and R132T. Both mutants exhibited infectivity similar to the wild type virus (data not shown). Therefore, the second-site mutations function by relieving a defect in a step of the virus infection cycle prior to integration.

### The T216I mutations partially relieves the reverse transcription impairment exhibited by P38A

**Figure 3:**
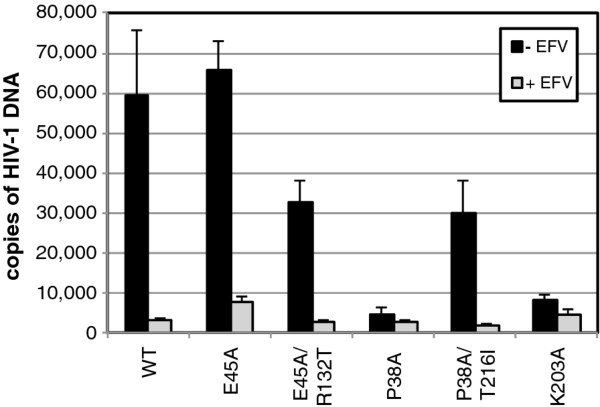
**Quantitative analysis of reverse transcription of CA mutants in target cells**. HeLa-P4 cells were inoculated with the indicated HIV-1 mutants, and the second-strand transfer viral DNA products accumulated at 8 hours were quantified by PCR. In half of the samples, the reverse transcriptase inhibitor Efavirenz (EFV) was included as a control for plasmid DNA contamination carryover from the transfections used to produce the virus stocks. Error bars represent the standard deviations of triplicate infections, and the results are representative of three independent experiments.

Our group previously showed that the P38A and E45A mutant capsids exhibited altered stability without major structural defects, and were impaired for reverse transcription, suggesting that the mutations result in aberrant uncoating in target cells [17]. Accordingly, the P38A substitution destabilized the viral capsid, while E45A resulted in a hyperstable capsid. In follow-up studies, we have repeatedly observed that the E45A mutation does not impair reverse transcription, but results in impairment at later stages of infection, after nuclear entry (Figure 3). In this respect, E45A resembles the Q63A/Q67A CA mutant, which exhibits delayed uncoating in target cells and is impaired for nuclear import and integration [16,17]. Importantly, the T216I mutation partially rescued the impaired reverse transcription exhibited by P38A (Figure 3), consistent with the interpretation that the impaired infectivity exhibited by P38A is owing to its reduced reverse transcription capacity. Collectively, these results suggest that a stable capsid is necessary for efficient reverse transcription in target cells, while mutants with hyperstable capsids are impaired at later steps of infection.

### Second-site suppressor mutations do not restore normal capsid stability in vitro

**Figure 4:**
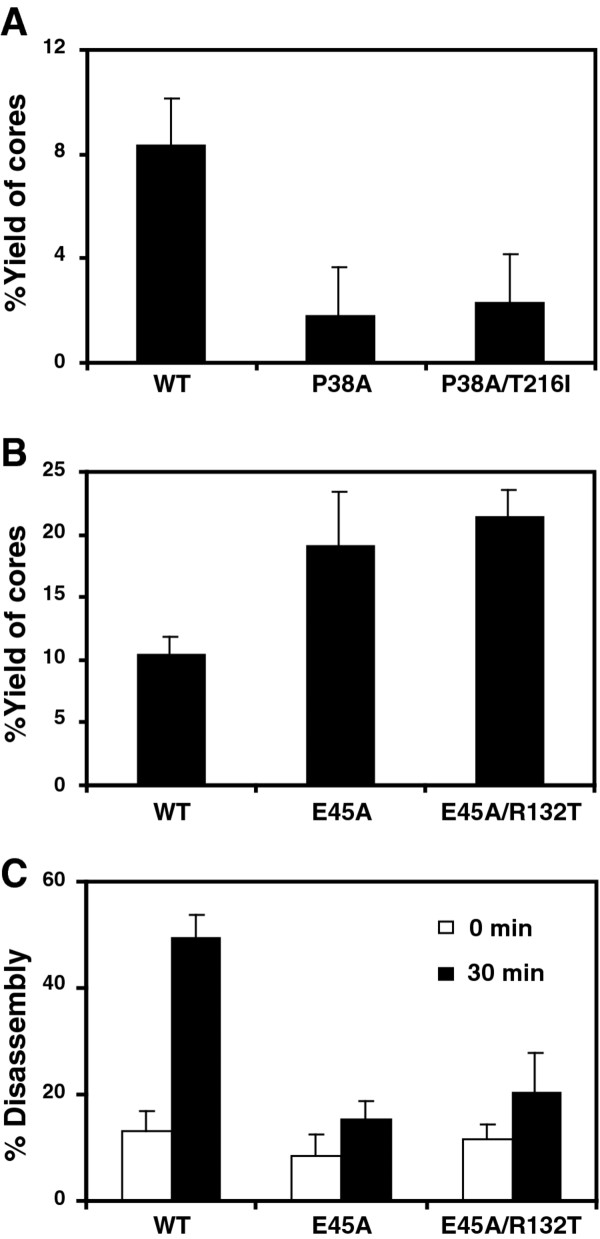
**Second-site suppressor mutations do not restore capsid stability *in vitro***. (A and B) Concentrated virions were subjected to ultracentrifugation through a detergent layer into a sucrose density gradient. Yields of cores were calculated as the percentage of the total CA that was detected in the peak fractions of cores. Results shown are the mean values of three independent experiments, with error bars representing one standard deviation. (C) Disassembly of purified HIV-1 cores *in vitro*. Diluted HIV-1 cores were incubated at 37°C for the indicated times, followed by separation of free and core-associated CA by ultracentrifugation. Supernatants and pellets were analyzed by p24 ELISA. The extent of disassembly was determined as the percentage of the total CA protein in the reaction detected in the supernatant. Results shown are the average values of two independent experiments with duplicate determinations in each experiment. Error bars represent the spread of values obtained in the two experiments.

To determine whether the compensatory mutations restore normal stability to the viral capsid, we isolated cores from each of the mutant viruses by centrifugation through a layer of nonionic detergent into a linear sucrose gradient. We quantified the recovery of core-associated CA in the fractions corresponding to the viral cores as a percentage of the total quantity of CA in the entire gradient. This value was approximately 10% for wild-type HIV-1 (Figure 4A and 4B). In contrast, the recovery of CA in P38A cores was about 2%, while that for E45A cores was approximately 20% (Figure 4B). Contrary to our expectation, cores recovered from the double mutants P38A/T216I and E45A/R132T exhibited CA levels that were similar to those of the corresponding single mutants (Figure 4A and 4B). To further evaluate the relative stability of E45A mutant HIV-1 cores, we assayed the rate of CA dissociation from purified cores in vitro. The poor recovery of core-associated CA from the P38A mutant particles precluded its analysis. Cores isolated from E45A and E45A/R132T both exhibited slower uncoating in vitro relative to wild-type cores (Figure 4C). Collectively, these results indicate that the suppressor mutations do not correct the aberrant intrinsic stability of the P38A and E45A mutant capsids observed in vitro.

### Suppressor mutations do not restore normal CA assembly kinetics *in vitro*

**Figure 5:**
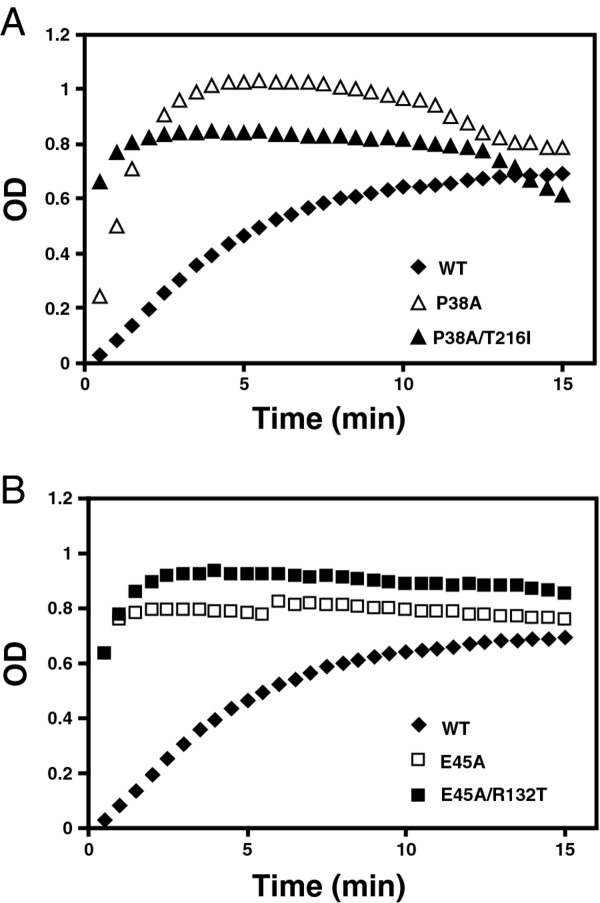
***In vitro *capsid assembly analysis**. To initiate assembly, purified recombinant CA proteins were diluted into a buffer, resulting in a final NaCl concentration of 2.25 M. The turbidity of the samples was determined with a spectrophotometer at the indicated times. For reference, the results for the wild type CA protein are shown in both panels A and B. Values shown are the average of two parallel determinations. Shown are data from one representative of 3 independent experiments.

Purified HIV-1 CA protein can assemble into tube-like structures in a high salt buffer system in vitro [27-30]. The E45A mutation was previously reported to accelerate HIV-1 CA assembly in vitro [31], suggesting that this mutation enhances protein-protein interactions between CA subunits. Reasoning that the hyperstability of the E45A viral capsid may be related to the accelerated CA assembly kinetics *in vitro*, we asked whether the suppressor mutations would affect the rate of CA assembly. Purified recombinant CA proteins encoding the E45A and E45A/R132T mutations were diluted into a high salt buffer, and the turbidity of the solution was monitored spectrophotometrically for 15 minutes following initiation of the reactions. The wild-type CA proteins assembled at a moderate rate, taking nearly 10 minutes for completion. In contrast, the E45A mutant assembled within 2 minutes (Figure 5A). However, the accelerated rate of assembly of E45A was not affected by the suppressor mutation R132T. The P38A and P38A/T216I mutant proteins also exhibited accelerated assembly, reaching completion within 3-4 minutes (Figure 5B). Thus, the effects of the E45A and P38A mutations on CA assembly in vitro were not correlated with the biological phenotypes of the corresponding CA mutant viruses.

### Structural assessment of mutant CA^151 ^proteins by NMR

**Figure 6:**
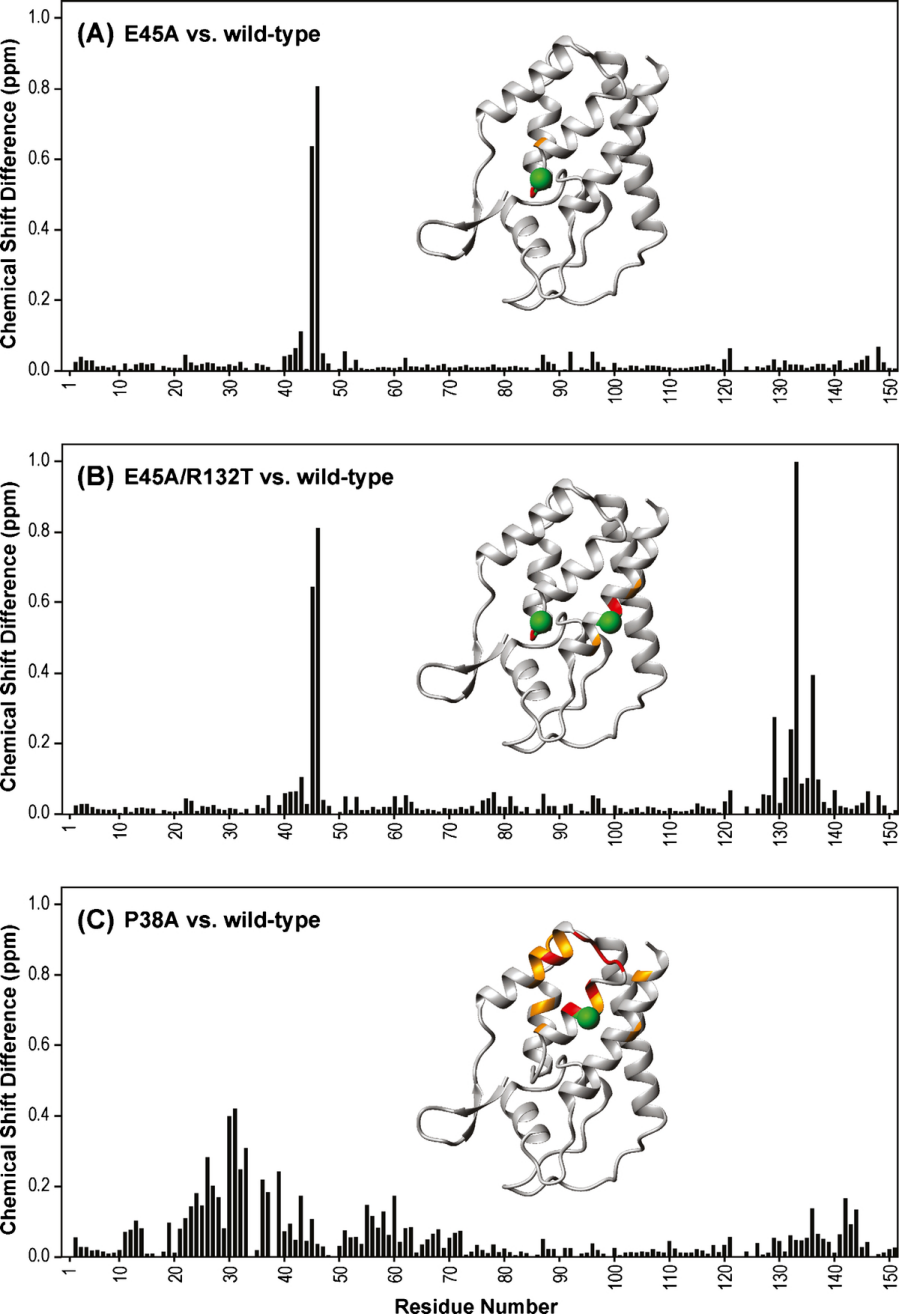
**Amide chemical shift differences between the wild type and E45A, E45A/R132T and P38A mutant CA-NTD proteins**. Combined ^1^H, ^15 ^N chemical shift differences between wild-type and mutants E45A (A), E45A/R132T (B) and P38A (C) are plotted versus residue number. Structural mapping of the chemical shift differences onto the X-ray structure of CA-NTD [1AK4] in ribbon representation is provided in the inset. The locations of the mutation sites are marked with large green spheres. Positions of residues whose amide resonances exhibit significant chemical shift differences are color-coded according to the magnitude of the change: red, Δδ > (Δδ_average _+ 2 × SD); orange, (Δδ_average _+ 2 × SD) > Δδ > (Δδ_average _+ 1xSD).

^1^H-^15 ^N HSQC NMR experiments were performed to assess the effects of the E45A, P38A, E45A/R132T and R132T mutations on the structure of the N-terminal domain of CA (CA-NTD). Superpositions of the ^1^H-^15 ^N HSQC spectra of wild-type and E45A (A), P38A (B), E45A/R132T (C) and R132T (D) mutants are shown in Additional file 1. The spectra of all the mutants are very similar to that of wild type, demonstrating conservation of the overall global fold. Chemical shift changes due to the mutations are plotted versus residue number in Figure 6. Resonance changes caused by the E45A and R132T mutations when compared with the wild type spectrum were essentially confined to the mutation sites. For example, in the E45A mutant only Gly46 (except the mutated residue Glu45) exhibited a large (~ 0.8 ppm) chemical shift change. Similarly, only neighboring residues (Ile129, Trp133, and Leu136) were associated with noticeable changes, ranging from 0.3 to 1 ppm in the R132T mutant. In contrast, the spectrum of the P38A mutant exhibited overall smaller changes (< 0.42 ppm), but the mutation affected more residues that were dispersed over a wider region. For example, eighteen residues exhibit chemical shift changes larger than the average change plus one standard deviation (SD) (0.14 ppm). These residues are located around the mutation site (such as Va36, Ile37 and Met39 on helix α2), but also in the preceding loop (Lys30-Ser33), helices α1 (Trp23-Glu28), α3 (Met55) and α7 (L136 and V142). This suggests that a more general effect such as an overall loosening of the structure may have occurred. We conclude that the E45A and R132T mutations induce highly localized structural changes, indicating that the effects of the mutations simply result from the change in chemical nature of the substituted amino acid. The small, but more widespread effects of the P38A mutation on the protein structure suggest that modulation of structural properties occurs in the mutant. Thus, the reduced capsid stability of the mutant may result from subtle changes in the overall structure and from Pro38's involvement in the NTD-NTD interaction in the assembled capsid. Note that Pro38 is in close proximity (< 5A) to Thr54, Asn57, and Thr58 of the neighboring CA molecule in the CA hexamer structure (3H47)[10].

### The R132T mutation relieves the cell-cycle dependence of the E45A mutant

**Figure 7:**
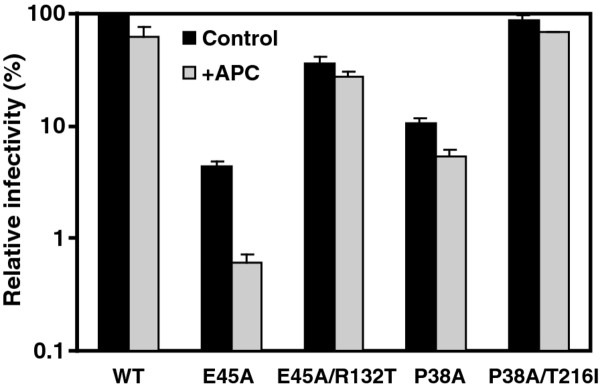
**Second-site suppressor mutation R132T relieves the cell-cycle dependence of the E45A mutant in a single-cycle infectivity assay**. Control and aphidicolin-arrested HeLa-P4 cells were inoculated with the indicated viruses. Infectivity was determined as described in the legend to Figure 2. Results shown are from one representative of three independent experiments.

HIV-1 efficiently infects both dividing and nondividing cells, and the capacity to infect nondividing cells has been linked genetically to CA [32]. Several mutations in CA, including E45A, result in selective impairment of infection of mitotically-arrested cells [25]. We therefore asked whether introduction of the R132T mutation in the E45A mutant virus CA would relieve its cell-cycle-dependence in arrested HeLa cells. Infection by the E45A mutant was reduced by eight-fold in arrested vs. control cells, while the wild type and E45A/R132T double mutant were only slightly (less than 50%) reduced in arrested cells. Thus, the R132T second-site substitution rescued the impaired ability of E45A infection in nondividing cells. In contrast, the P38A and P38A/T216I mutants behaved similar to wild type HIV-1 and were only slightly less infectious for arrested vs. dividing cells (Figure 7). These results indicate that the R132T mutation corrects the selective impairment of infection of nondividing cells associated with the E45A mutant virus.

### Addition of the R132T substitution restores sensitivity of the E45A mutant to the small molecule inhibitor PF74

**Figure 8:**
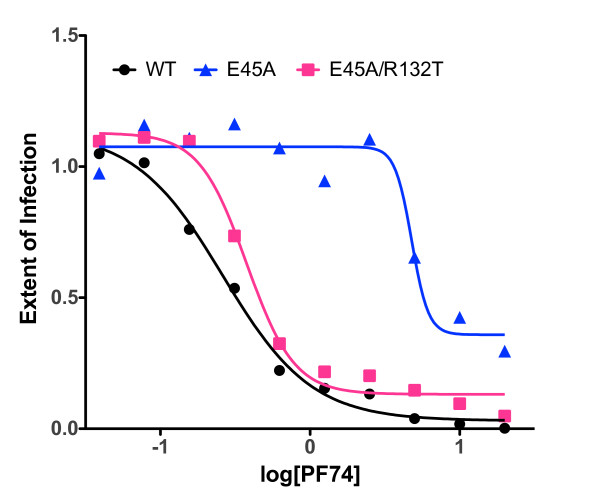
**Inhibition of wild type, E45A, and E45A/R132T mutant HIV-1 by PF74**. Viruses were added to HeLa-P4 target cells at predetermined dilutions in the presence of the indicated concentrations of PF74. Infected cells were identified two days later by X-gal staining and quantified. Results were expressed as the extent of infection normalized by the values obtained with the no-drug controls.

In a recent study, we showed that the small molecule HIV-1 inhibitor PF74 binds and destabilizes the viral capsid, leading to premature HIV-1 uncoating in target cells [33]. The E45A substitution rendered HIV-1 resistant to PF74, presumably by stabilizing the capsid, as the mutation did not reduce PF74 binding to mature HIV-1 particles. To further probe the effects of the suppressor mutation R132T on the phenotype of the E45A mutant in target cells, we compared the sensitivity of wild type, E45A, and E45A/R132T mutant viruses to inhibition by PF74 in single-cycle infection assays. The compound was added at various doses to the cells at the time of virus inoculation, and the number of infected cells quantified after two days of culture. As previously shown, the E45A mutant virus exhibited strong resistance to PF74 (Figure 8). By contrast, the E45A/R132T mutant exhibited nearly wild type sensitivity (IC50 = 0.38 μM vs. 0.26 μM for the wild type virus). The restored sensitivity of the double mutant virus to PF74 suggests that the R132T mutation partially reverses the E45A-induced uncoating defect in target cells.

### The T216I mutation restores the ability of P38A particles to saturate TRIM5 host restrictions in monkey cells

The host tripartite motif proteins TRIMCyp and TRIM5α act as barriers to lentivirus infection by targeting the viral capsid in a species-specific manner [34-39]. Restriction of HIV-1 by TRIM5 proteins in simian cells can be saturated by adding virus-like particles (VLPs) *in trans*. In previous work, we showed that the ability of HIV-1 VLPs to overcome restriction by TRIM5α and TRIMCyp is reduced by CA mutations that destabilize the viral capsid [40,41], but not by mutations that render the capsid hyperstable, leading us to conclude that efficient *trans*-abrogation of TRIM5 restriction requires particles with a stable capsid. The ability to abrogate restriction *in trans *does not depend on the infectivity of the decoy particles, as poorly infectious mutants containing hyperstable capsids (e.g. E45A) were competent in the assay. Our previous results from the assay also supported the conclusion that HIV-1 CA mutants containing unstable capsids undergo accelerated uncoating in target cells. Therefore, the dependence of trans-abrogation activity on capsid stability provides an informative probe of capsid disassembly in target cells.

**Figure 9:**
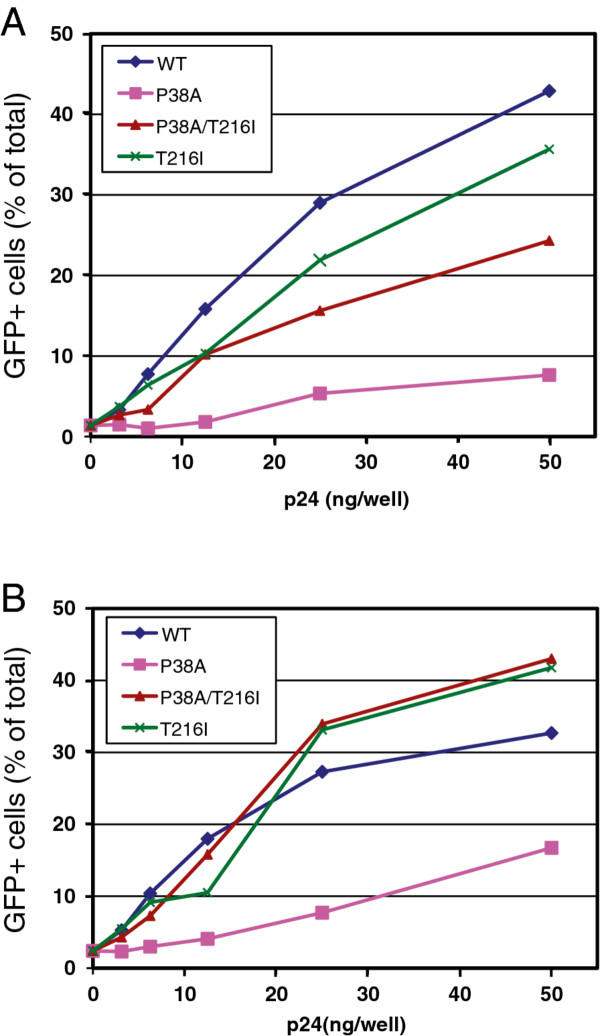
**The second-site mutation T216I restores the ability of P38A particles to saturate TRIM5 restrictions in simian cells**. Cultures of OMK (A) and FRhK-4 (B) cells were inoculated with the indicated quantities of VSV-G-pseudotyped CA mutant HIV-1 particles and a fixed subsaturating quantity of GFP-encoding pseudotyped HIV-1 particles. Results shown are representative of two independent experiments.

In previous studies, we observed that the P38A mutation impairs the ability of HIV-1 particles to abrogate host restriction in simian cells owing to capsid instability. To test whether the second-site suppressor restores capsid stability in target cells, we asked whether T216I restores the ability of P38A particles to abrogate restriction. Owl monkey (OMK) cells, which express TRIMCyp, were inoculated with a fixed quantity of HIV-GFP reporter virions with various quantities of wild type or mutant HIV-1 particles (non-GFP-encoding). Titration of the wild-type particles led to a marked enhancement of infection by the reporter virus (Figure 9A). By contrast, P38A mutant particles were approximately 80% less effective at abrogating restriction, as we previously reported [40]. For P38A/T216I mutant particles, the activity was intermediate between wild type and P38A levels. In rhesus macaque cells, in which HIV-1 infection is restricted by TRIM5α, the impaired abrogation activity of P38A mutant particles was also rescued by the T216I suppressor mutation (Figure 9B). In both cell lines, the T216I single mutant exhibited a similar level of activity as the wild type, demonstrating that the rescue of the P38A mutant activity was not due to a general enhancement of activity by the suppressor mutation. Collectively, these results suggest that the T216I mutation prevents premature disassembly of the P38A mutant core in target cells, thereby relieving the impaired ability of the mutant core to interact with restriction factors.

## Discussion

Uncoating, defined as disassembly of the viral capsid in target cells, is a critical yet poorly understood stage of retroviral infection. In the present work, we observed that the R132T and T216I mutations can partially correct the infectivity defects associated with E45A and P38A, respectively, without correcting the altered capsid stability perturbations created by the original mutations. Similar observations have been reported for RSV, where second site suppressors of CA mutants failed to correct aberrant capsid stability [6]. To elucidate the mechanisms by which the suppressor mutations rescue the original mutant phenotypes, we examined the kinetics of CA protein assembly *in vitro*, and performed target cell-based assays for viral functions that depend on HIV-1 capsid stability. CA assembly assays did not uncover any correlation between capsid stability and the mutant phenotypes. The cell-based assays, however, were more revealing. The T216I mutation reversed the impaired ability of the P38A mutant to abrogate TRIMCyp- and TRIM5α-mediated restriction of HIV-1. We previously demonstrated a correlation between efficiency of saturation of restriction and intrinsic capsid stability [40,41]; therefore, the restored ability of P38A/T216I to saturate CA-specific host restriction likely results from stabilization of the viral capsid in the target cells. Furthermore, the R132T mutation partially rescued the impaired ability of the E45A mutant to infect nondividing cells, possibly via reversal of the uncoating defect caused by the E45A mutation. Similarly, the R132T suppressor restored sensitivity to PF74, a compound that inhibits HIV-1 by destabilizing the viral capsid. Collectively, these data strongly suggest that the E45A uncoating defect is rescued by the R132T mutation. Apparently, the putative uncoating defect associated with the P38A and E45A mutations can be rescued in target cells without restoring normal intrinsic capsid stability.

Although uncoating has not been definitively linked to the ability of lentiviruses such as HIV-1 to infect nondividing cells, recent studies demonstrate a role of the CA protein [25,32], and completion of uncoating may be a requirement for mitosis-independent infection. In accordance with this hypothesis, impaired infection of nondividing cells is associated with CA mutants E45A and Q63A/Q67A, which exhibit hyperstable capsids and/or slower uncoating in target cells. Our observation that R132T rescues the impaired ability of E45A mutant particles to infect arrested cells confirms that the ability of lentiviruses to infect nondividing cells is dependent on a function of the viral capsid, and suggests that this is linked to the mechanism or timing of uncoating.

**Figure 10:**
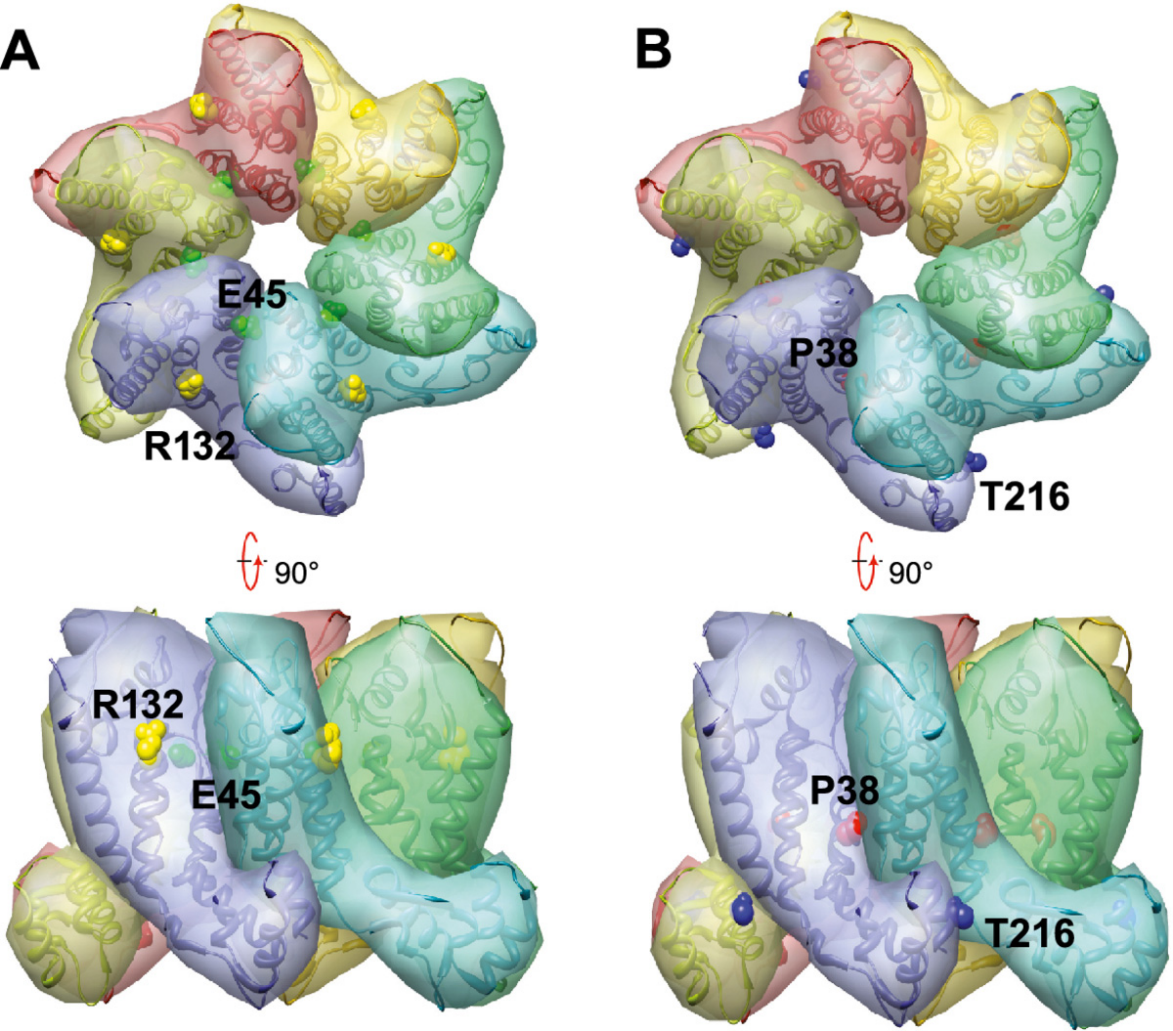
**Locations of mutations in the structure of the CA hexamer**. (A) Top and side views of a CA hexamer showing E45 (green) and R132 (yellow) side chains from the NMR structure of CA^151 ^(PDB ID: 3H47). (B) Same views, highlighting P38 and T216. P38 (red) in helix 2 is located at the CA NTD-NTD interface and is adjacent to helix 3 in the adjacent subunit. T216 (blue) in helix 11 is near the CA NTD-CTD interface and is close to helix 7 in the nearby subunit.

Our NMR structural analysis of CA-NTD proteins suggests that the mutant phenotypes are not a consequence of major structural alterations in the capsid. NMR structural assessment of the effects of the mutations on the CA-NTD structure revealed no gross conformational changes in either P38A or E45A mutants, nor for the R132T mutant. The P38A substitution, however, resulted in more extensive changes that could be related to a general effect of the structural adjustment for the mutation. Examination of the locations of these amino acids in recently reported structures of the HIV-1 CA hexamer [9,10] revealed that R132 is positioned on the surface of the CA molecule facing the NTD-NTD interface (Figure 10A). E45 is in the vicinity of this interface as well; however, the distance between these two side chains prohibits a direct interact with one another, making it unlikely that R132T directly offsets the stability defect resulting from E45A. The R132T substitution could act by restoring the overall net charge of CA, which would be consistent with our previous findings that the stability of the HIV-1 capsid is sensitive to changes in ionic strength and pH [17]. Alternatively, the E45A mutation might affect the network of water-mediated hydrogen bonds observed at the NTD-NTD interface in the X-ray crystal structure [10], with R132T mutation partially restoring the H-bonding network. It should be noted that the hexamer crystal structure was determined with a mutant protein that contained two amino acid substitutions, one of which was a Glu to Cys change position 45.

Our results with the P38A/T216I mutant are reminiscent of another study of CA-NTD mutants in which a second-site substitution at position 208 in the CTD restored replication to mutants containing substitutions at His62 in the NTD, which were associated with structural impairments in the capsid [42]. A recent cryoEM structural model of the assembled capsid may help explain how T216I, which resides in the C-terminal domain of CA, modulates the effects of the P38A substitution in the N-terminal domain. In the model, T216 resides at the CTD-CTD interhexamer interface. Substitution of Cys at positions 207 and 216 resulted in intersubunit disulfide crosslinking within HIV-1 particles, supporting the model [43]. Thus, the T216I substitution may stabilize the CTD-CTD interhexamer interface, thereby offsetting the structural effects of the P38A substitution. Moreover, T216 is also near the NTD-CTD intersubunit interface in the hexamer (Figure 10B), where it may also modulate the stability of the viral capsid.

In our previous studies, we observed a strong dependence of HIV-1 infection on the proper stability of the viral capsid. Specifically, ten CA mutants exhibiting altered capsid stability were impaired for infectivity, while five other mutants with normal infectivity exhibited capsid stability similar to the wild type [17]. This begs the question: how do the CA suppressor mutations rescue HIV-1 infectivity without restoring normal capsid stability in vitro? While the simplest interpretation is that capsid stability is irrelevant for infection, we think this improbable because of the strong correlation we previously observed. One possible alternative explanation is that the biochemical assays we have employed are limited in their ability to detect subtle changes in capsid stability. HIV-1 capsid stability is altered by changes in pH and ionic strength *in vitro *[17], and the conditions in which uncoating takes place in target cells are unknown. A second possibility, which we favor, is that the suppressor mutations alter interactions with host factors that participate in HIV-1 uncoating in target cells. Based on the current and previous *in vitro *and cell-based studies, we propose that HIV-1 infection is dependent on the proper HIV-1 capsid stability, but that the effects of changes in intrinsic capsid stability can also be mitigated by secondary mutations that alter interactions with host cell molecules, or their consequences. In support of this hypothesis, we have observed that an HIV-1 CA mutant with five substitutions in CA, remains infectious despite exhibiting a remarkably stable capsid [33,44]. Furthermore, the observation that HIV-1 can escape requirement for expression of host factor point mutations in CA confirms that CA exhibits functional plasticity [45]. Additional studies of the determinants of HIV-1 capsid stability and its interactions with host factors should lead to an improved understanding of HIV-1 uncoating and facilitate the discovery and development of pharmacologic inhibitors targeting this critical stage of the virus life cycle.

## Conclusions

We have identified second-site suppressors of mutations that alter the stability of the viral capsid. The suppressors restore the ability of the mutant viruses to replicate in T cells, and revert the mutant phenotypes in several cell-based functional assays. However, the biochemical defects in capsid stability produced by the original mutations were not corrected by the suppressor mutations. We propose that proper uncoating in target cells depends on the intrinsic stability of the capsid and on interactions between the viral capsid and host cell factors.

## Methods

### Cells and viruses

293 T, HeLa-CD4/LTR-lacZ (Hela-P4) cells were cultured in Dulbecco's modified Eagle's medium (Cellgro) supplemented with 10% fetal bovine serum, penicillin (50 IU/ml), and streptomycin (50 μg/ml) at 37°C and 5% CO_2_. CEM cells were cultured in RPMI 1640 medium with the same supplements. The wild-type HIV-1 proviral DNA construct R9 [46], encoding full-length open reading frames for all HIV-1 structural and accessory genes, was used for these studies. R9 clones encoding mutations E45A and P38A in CA were previously described [47] and were the generous gift of Dr. Wes Sundquist. HIV-GFP, an *env*-defective proviral clone encoding GFP in place of *nef *[48], was used to generate pseudotyped reporter particles. Viruses were produced by calcium phosphate transfection of 293 T cells (20 μg of plasmid DNA per 2 × 10^6 ^cells) as previously described [49]. Five μg of pHCMV-G [50] were included in the transfection mix for producing VSV-G-pseudotyped viruses with *env*-defective variants of the respective proviral clones. One day after transfection, culture supernatants were harvested and clarified by passage through 0.45-μm-pore-size filters, and aliquots were frozen and stored at -80°C. The CA concentration of each virus stock was quantified by p24 enzyme-linked immunosorbent assay (ELISA), as previously described [51]. Reverse transcriptase activity was quantified in viral stocks by polymerization of ^3^H-TTP, as previously described [52].

### HIV-1 replication assay

Viral replication was determined by inoculation of cultures of CEM cells (2 × 10^5 ^cells in 0.2 ml) with quantities of virus corresponding to 2 ng of p24 antigen. Every two days, samples of culture supernatants (100 μl) were withdrawn and replaced with an equal volume of fresh medium. Reverse transcriptase activity in culture supernatants was quantified as previously described [53].

### HIV-1 adaptation studies

Virus stocks, normalized for RT activity, were used to inoculate CEM cell cultures. Culture supernatants were harvested at peak of RT activity, and normalized quantities of viruses were re-inoculated in to new CEM cultures. The mutant viruses emerged after approximately one month of culture. Two additional passages of infection in fresh CEM cells were performed until accelerated growth (emergence in 15-20 days) was observed. Cells were harvested immediately following the peak of RT activity from the third passage. Proviral DNA was purified with the DNeasy kit (Qiagen), and regions of Gag were PCR amplified using *pfu *DNA polymerase (Stratagene) to a 800-bp *BssH*II-*Spe*I fragment spanning MA and part of CA coding region and a 500-bp *Spe*I-*Apa*I fragment spanning part of CA and NC coding region. The *BssH*II-*Spe*I fragment was amplified using sense primer 5'-GGAGATCTCTCGACGCAG-3' and anti-sense primer 5'- TTTAATCCCAGGATTATCCAT-3'. The *Spe*I-*Apa*I fragment was amplified using sense primer 5'-GCATGCAGGGCCTATTGC -3' and anti-sense primer 5'- CCTGTCTCTCAGTACAATC-3'. Following identification of additional mutations by DNA sequencing of the PCR products, the corresponding *Spe*I-*Apa*I fragments were transferred into R9.E45A or R9.P38A proviral clones. The individual single R132T and T216I mutants were constructed by transferring the mutant *Spe*I-*Apa*I segments into the wild-type R9 plasmid. Mutant viral constructs were verified by sequencing of the corresponding regions.

### Assay of HIV-1 infectivity

The HeLa-P4 cell line, a HeLa cell clone engineered to express CD4 and an integrated long terminal repeat (LTR)-*lacZ *reporter cassette [54], was used to quantify HIV-1 infectivity, as previously described [53]. Infected cells were identified by staining with X-gal, and the specific infectivity of each virus was defined as the number of infected cells per ng of p24 antigen in the inoculum. PF74 was synthesized by the Vanderbilt Institute of Chemical Biology Synthesis Core, Vanderbilt University, Nashville, TN 37212. Assays of PF74 inhibition were performed with a fixed concentration of each virus, with the inhibitor present at the indicated concentrations in the inocula.

### Assay of HIV-1 uncoating *in vitro*

The uncoating of purified HIV-1 cores was assayed as described previously [17,55]. Purified cores were diluted in STE buffer and incubated at 37°C. Following incubation, the particles were subjected to ultracentrifugation to separate free CA from intact cores. Supernatants and pellets were analyzed for CA content by ELISA to determine the percentage of total CA released from the cores during the incubation.

### Saturation-of-restriction assays

VSV-pseudotyped HIV-GFP reporter particles were titrated onto FRhK-4 and OMK cell monolayers to determine the appropriate dose for use in the assays. The abrogation-of-restriction assay was performed as previously described [34,40]. Cultures (20,000 cells per well in 24-well plates) were inoculated with fixed (subsaturating) quantity of an HIV-GFP reporter virus together with various concentrations of VSV-G-pseudotyped non-reporter viruses in the presence of polybrene (8 μg/ml) in a total volume of 300 μl. One day later, complete medium (1 ml) was added. Two days after infection, cells were detached using trypsin and were fixed by the addition of an equal volume of phosphate-buffered saline containing 4% paraformaldehyde. Cellular GFP expression was quantified by flow cytometry by using a FACSCalibur instrument (Becton Dickinson), and the percentage of GFP-expressing cells was determined with Cellquest software. A minimum quantity of 5,000 cells was analyzed for each sample.

### CA protein expression and purification

pET21a-based plasmids were constructed for full-length CA protein expression in *E. coli *BL21-DE3. An NdeI-XhoI fragment was amplified from WT and mutant CA cDNA using sense primer 5'- GGAATCCCATATGCCTATAGTGCAGAACCTCCAGGGG-3' and anti-sense primer 5'- CCCCTCGAGTCACAAAACTCTTGCTTTATGGCCGGG-3' and inserted into the NdeI-XhoI site of pET21a and verified by DNA sequencing. CA proteins were expressed and purified as previously described [56], except where noted. Proteins were eluted from a UnosphereQ (Bio-Rad) column at NaCl concentrations ranging from 70 to 90 mM. Most of the E45A protein was present in the flow-through. The protein solutions were dialyzed into 50 mM sodium phosphate buffer, pH 8.0, concentrated with Centriprep centrifugal filters (Millipore) to a concentration of ~300-500 μM and stored frozen at -80°C until needed. The proteins were > 99% pure by SDS-PAGE.

To produce isotopically-labeled CA proteins for NMR studies, cells were grown in modified minimal medium at 23°C using ^15^NH_4_Cl and/or ^13 ^C glucose as a sole nitrogen and carbon source in *E. coli *Rosetta 2 (DE3) and induced for 16 h with 0.4 mM IPTG. Soluble CA-NTD proteins were obtained by sonication in lysis buffer containing 25 mM sodium phosphate, pH 7.0, 1 mM DTT, and 0.02% sodium azide followed by ultracentrifugation at 127,000 × *g*. Proteins were purified by using 10 mL Hi-Trap QP (GE Healthcare, Piscataway, NJ) at pH 7.0 and pH 8.5 with 1 M NaCl gradient. The fractions containing CA-NTD were further purified using Hi-Load Superdex75 26/60 (GE Healthcare, Piscataway, NJ) equilibrated with 25 mM sodium phosphate, pH 6.5, 100 mM NaCl, 1 mM DTT, and 0.02% sodium azide. The molecular mass of each CA-NTD protein was confirmed by LC-ESI-TOF mass spectrometry (Bruker Daltonics, Billerica, MA) and isotope labeling efficiency was estimated to be greater than 99%.

### Assays of CA assembly *in vitro*

Purified full-length CA proteins were assembled by rapid dilution into concentrated NaCl solutions at 23°C to yield a final concentration of 2.25 M NaCl and protein of 50 μM, and the course of the reaction was monitored by turbidity. For kinetic analysis, the reaction was rapidly mixed and placed into a 1-mm quartz cuvette. Approximately 20 s elapsed between the time of the addition of salt and the first time point measured. The increase in optical density was monitored at 350 nm for 30 min as previously described [31].

### NMR analysis

Experiments were conducted at 25°C on U-[^15 ^N,^13 ^C]- or U-[^15 ^N]-labeled CA-NTD samples in NMR buffer (25 mM sodium phosphate buffer, pH 6.5, 100 mM NaCl, 2 mM DTT, and 0.02% sodium azide) using Bruker Avance 700 and 600 MHz spectrometers, equipped with 5 mm, triple resonance and z-axis gradient cryoprobes. Complete backbone assignments of the wild-type CA-NTD were obtained using 3D HNCACB and HN(CO)CACB experiments on U-[^13 ^C,^15 ^N]-labeled proteins. Complete assignments of the 2D ^1^H-^15 ^N HSQC spectra for the E45A, R132T and E45A/R132T mutant proteins were obtained unambiguously by direct comparison with the spectrum of wild-type protein since the spectra of the mutants are essentially identical to that of wild-type, except for few resonances arising from amino acids around the mutation sites. The assignment of the P38A mutant spectra were less straightforward since more (~ 20) resonances exhibited changes > 0.1 ppm from the wild type. Nevertheless, all but two resonances (Trp23 and Met55) were unambiguously assigned. Structural perturbations created by the mutations were assessed by evaluating the combined amide chemical shift differences (Δδ) between wild-type and mutant using the equation

$$\Delta \delta =\surd \left({\left(0.1*\Delta \delta_{\text{N}}\right)}^{2}+\Delta \delta_{\text{H}{\text{N}}^{2}}\right),$$

with Δ*δ_HN _*and Δ*δ_N _*representing the ^1^HN and ^15 ^N chemical shift differences, respectively.

### Cell cycle arrest

To generate cell cycle-arrested target cells for infectivity assay, HeLa-P4 cells (4 × 10^4 ^cells/well) were plated in 48-well plates. Aphidicolin (Sigma-Aldrich) was added to a final concentration of 2 μg/ml upon seeding [25,57]. The next day, cells were inoculated with viruses overnight; aphidicolin was also present during this period. Cultures were replenished with 0.5 ml fresh media and cultured for another 24 hours prior to X-gal staining to reveal infected cells.

### Assays of HIV-1 proviral DNA in target cells

HIV-1 stocks were treated with DNaseI, and quantities corresponding to 100 ng p24 were used to inoculate cultures of HeLa-P4 cells (100,000) in triplicate, in the presence or absence of the reverse transcriptase inhibitor Efavirenz (1 μM). Total DNA was harvested at 8 hours post-inoculation and was purified using the DNeasy kit (Qiagen). DNA was eluted in 100 μl of water. Five microliter aliquots were assayed for 2^nd^-strand transfer reverse transcription products by quantitative PCR, as previously described [17].

### Structural representation

Molecular graphics images in Figure 6 were produced with MOLMOL [58], and those in Figure 9 were produced using the UCSF Chimera package from the Resource for Biocomputing, Visualization, and Informatics at the University of California, San Francisco (supported by NIH P41 RR-01081)[59]. The images are based on the 1.9A resolution structure of the CA hexamer (Protein Data Bank entry 3H47.pdb)[10].

## Abbreviations

HIV-1: Human immunodeficiency virus type 1; NTD: Amino-terminal domain; RNP: Ribonucleoprotein; PIC: Preintegration complex; CTD: Carboxy-terminal domain; NMR: Nuclear magnetic resonance; RSV: Rous sarcoma virus; VLPs: Virus-like particles; DTT: Dithiothreitol; VSV-G: Vesicular stomatitis virus glycoprotein.

## Competing interests

The authors declare that they have no competing interests.

## Authors' contributions

RY, AMG, and CA conceived and designed the experiments; RY, JS, I-JLB, and JA performed the experiments; JHS and JM produced the structural figures; RY, I-JB, JA, AMG, and CA wrote the manuscript. All authors read and approved the final manuscript.

## Supplementary Material

Additional file 1 **Figure showing monitoring of structural perturbations of CA-NTD caused by mutations with 1H-15 N HSQC NMR**. Superimposed 1H-15 N HSQC spectra of 15 N-labeled wild-type (black) and mutants (red), E45A (A), P38A (B), E45A/R132T (C) and R132T (D). The peaks showing substantial perturbation due to the mutation are labeled with assignments. Click here for file
