# Supplementary material for: Second-site suppressors of HIV-1 capsid mutations: restoration of intracellular activities without correction of intrinsic capsid stability defects
Source: Retrovirology. 2012 Apr 19;9:30. doi: 10.1186/1742-4690-9-30 (PMC3351742; doi:10.1186/1742-4690-9-30)
Supplement: Additional file 1 — Figure showing monitoring of structural perturbations of CA-NTD caused by mutations with 1H-15 N HSQC NMR. Superimposed 1H-15 N HSQC spectra of 15 N-labeled wild-type (black) and mutants (red), E45A (A), P38A (B), E45A/R132T (C) and R132T (D). The peaks showing substantial perturbation due to the mutation are labeled with assignments. [file 1742-4690-9-30-S1.PDF]

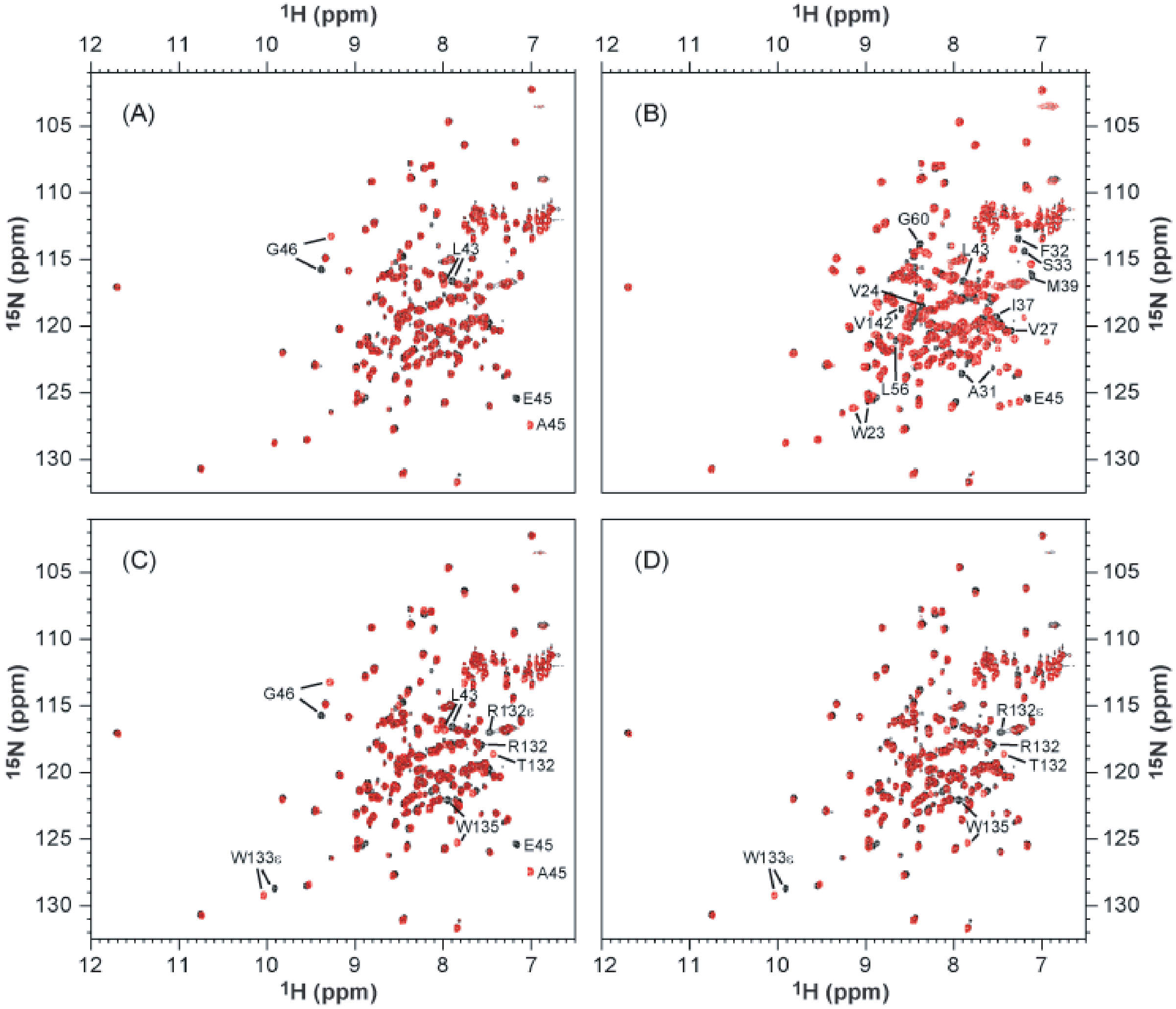

Information for Additional File 1: Figure showing monitoring of structural perturbations of CA-NTD caused by mutations with 1H-15N HSQC NMR. Superimposed 1H-15N HSQC spectra of 15N-labeled wild-type (black) and mutants (red), E45A (A), P38A (B), E45A/R132T (C) and R132T (D). The peaks showing substantial perturbation due to the mutation are labeled with assignments.
